# A review of factors influencing sensitive skin: an emphasis on built environment characteristics

**DOI:** 10.3389/fpubh.2023.1269314

**Published:** 2023-12-04

**Authors:** Xiangfeng Chen, Jing Wen, Wenjuan Wu, Qiuzhi Peng, Xiangfen Cui, Li He

**Affiliations:** ^1^Department of Dermatology, First Affiliated Hospital of Kunming Medical University, Kunming, China; ^2^The Centre for Modern Chinese City Studies, East China Normal University, Shanghai, China; ^3^Faculty of Land Resources Engineering, Kunming University of Science and Technology, Kunming, China; ^4^Faculty of Environmental Science and Engineering, Kunming University of Science and Technology, Kunming, China; ^5^Yunnan Institute of Skin Health, Kunming, China

**Keywords:** sensitive skin, influencing factors, socioeconomic attributes, behavioral characteristics, natural environment, built environment

## Abstract

**Background:**

Sensitive skin (SS) is a condition characterized by hyperreactivity. Impacting around 37 percent of the worldwide population and exerting an influence on the quality of life for affected individuals. Its prevalence rate has increased due to factors such as elevating stress levels and deteriorating environmental conditions. The exposome factors influencing SS have extended from demographic, biological attributes, and lifestyle to external environments. Built environments (BEs) have demonstrated as root drivers for changes in behaviors and environmental exposure which have the potential to trigger SS, but the review of the associations between BEs and SS is currently lacking.

**Objective:**

This review aims to achieve two primary objectives: (1) Examine exposome factors that exert influence on SS at the individual and environmental levels. (2) Develop a theoretical framework that establishes a connection between BEs and SS, thereby offering valuable insights into the impact of the built environment on this condition.

**Methods:**

An extensive literature search was carried out across multiple fields, including sociology, epidemiology, basic medicine, clinical medicine, and environmental research, with a focus on SS. To identify pertinent references, renowned databases such as PubMed, Web of Science, and CNKI were utilized.

**Results:**

SS is the outcome of interactions between individual attributes and environmental factors. These influencing factors can be categorized into five distinct classes: (1) demographic and socioeconomic characteristics including age, gender, and race; (2) physiological and biological attributes such as emotional changes, skin types, sleep disorders, and menstrual cycles in women; (3) behavioral factors, such as spicy diet, cosmetic use, alcohol consumption, and physical exercise; (4) natural environmental features, including climate conditions and air pollution; (5) built environmental features such as population density, green space availability, road network density, and access to public transportation, also have the potential to affect the condition.

**Conclusion:**

The importance of interdisciplinary integration lies in its ability to ascertain whether and how BEs are impacting SS. By elucidating the role of BEs in conjunction with other factors in the onset of SS, we can provide guidance for future research endeavors and the formulation of interventions aimed at mitigating the prevalence of SS.

## 1 Introduction

According to the World Health Organization’s projections, chronic non-communicable diseases (NCDs) are anticipated to account for a staggering 80% of annually global deaths by 2030 (1). This shift signifies a remarkable transition, with NCDs superseding infectious diseases as the primary drivers of the overall disease burden (2). Conditions such as obesity, cardiovascular diseases, and respiratory ailments are at the forefront of this transformative landscape.

Skin disease stands as one of the most prevalent human ailments (3), and many of them are also NCDs. Notably, an analysis conducted for the Global Burden of Disease Study in 2017 revealed that the rising population growth and aging demographics in China have led to a substantial disease burden associated with skin conditions (4). Among these conditions, sensitive skin (SS) represents a commonly encountered clinical symptom and sign, primarily manifesting on the facial region. When exposed to physical, chemical, and psychological stimuli, individuals often experience subjective sensations such as burning, tingling, itching, and tightness. Objective signs such as erythema, scales, and telangiectasia may or may not accompany these symptoms (5). In 2016, the International Pruritus Research Forum defined SS as the unpleasant sensation triggered by external stimuli that typically do not elicit skin symptoms (6). The etiology of SS remains elusive, potentially involving sensory nerve dysfunction (7), heightened vascular reactivity (8), barrier impairments (9), and immune-inflammatory mechanisms of the skin (10). Additionally, the surge in SS cases is associated with environmental pollution and unhealthy lifestyles. Globally, the prevalence of SS is estimated at 36.9% (11), with some regions reporting prevalence rate as high as 50–70%. The presence of SS may negatively impact the quality of life for affected individuals (12). Lower self-confidence, mental disorders (13) and the economic burdens resulting from its high prevalence and recurrent nature impose substantial hardships on patients.

The interconnection between health and the environment has garnered increasing attention, particularly in the realm of public health research concerning the built environments (BEs). The BEs encompass the man-made spaces in which individuals engage in daily activities, work and leisure (14). Empirical studies have extensively linked BEs to a range of health-related behaviors (15, 16), mental conditions (17, 18), NCDs such as obesity (19), cardiovascular disease (20), and respiratory disease (21). Despite the growing body of research, the relationship between the BE and SS remains understudied. Previous epidemiological investigations on SS predominantly focus on individual sociology and lifestyles of the population, including age, gender, dietary habits and cosmetic Usage as well as natural environmental stressors such as sun exposure, air pollution and temperature (22, 23). This review aims to address the aforementioned research gap by providing a comprehensive profile of the influencing factors at individual and environmental levels. Furthermore, this study also puts forward a theoretical framework to elucidate the potential pathways linking the BEs to SS based on the established mechanisms that underpin the associations between BEs and health (24, 25).

## 2 Search strategies and selection of studies

**Figure 1:**
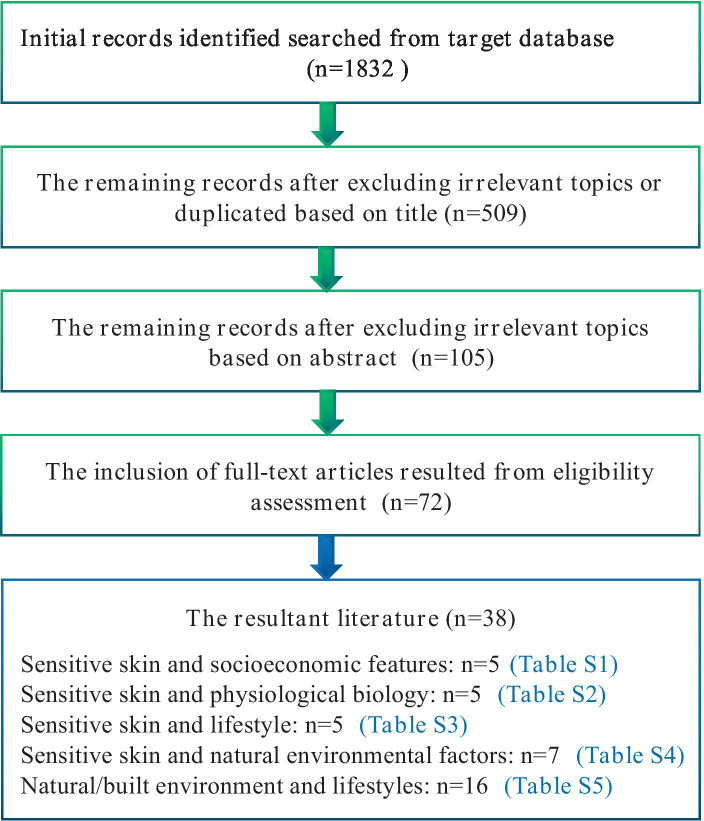
Flowchart for literature selection.

We conducted systematic searches in reputable databases, including PubMed, Web of Science, and CNKI (China National Knowledge Infrastructure), to identify relevant references published since 1997. The search strategy employed multiple combinations of the terms “sensitive skin” within the title/abstract, encompassing epidemiology, prevalence, socioeconomics, gender, ethnicity, skin type, emotional change, menstrual cycle, sleep, diet, spicy food, cosmetics, smoking, drinking, exposure, sun exposure, temperature, humidity, particles, pollution and the built environment. Our literature review did not uncover any studies directly addressing the built environment and SS. However, we did find evidence suggesting that the occurrence of SS is associated with exposure to natural environment, mental conditions and patterns of sleep and diet, all of which have been linked with BEs. Additionally, we checked the references cited in the identified papers to ensure the inclusion of all pertinent literature. The evaluation process involved assessing the title and abstract against our inclusion criteria. Subsequently, a full-text review was conducted to determine if the article met our standards. Inclusion criteria comprised studies written in English that focused on the epidemiology of SS. Studies that did not specifically focus on SS were excluded. The flowchart (Figure 1) depicted the process for the inclusion and exclusion of literature.

As illustrated in Figure 1, our literature review did not uncover any studies directly addressing the built environment and SS. However, we did find evidence suggesting that the occurrence of SS is associated with exposure to natural environment, mental conditions and patterns of sleep and diet, all of which have been linked with BEs. As a result, the scope of our search was broadened to incorporate the term “built environment” in conjunction with factors such as air pollution, sun exposure, mental conditions such as anxiety and depression, and lifestyle elements like sleep and diet (Figure 1). The influencing factors can be classified into five categories: (I) demographic and socioeconomic attributes; (II) physiological and biological characteristics; (III) lifestyles; (IV) natural environmental features; (V) built environmental features.

## 3 Demographic and socioeconomic attributes

### 3.1 Age

Research indicates a higher prevalence of SS in the young group than their elder counterparts. Notably, a French epidemiological survey comprising 5,000 individuals revealed that individuals under the age of 35 exhibit a higher prevalence of SS, surpassing 60% of the population within this age range (26). Similarly, a survey conducted in South Korea found that the younger group ranging from 15 to 44 years old displays a greater likelihood of developing SS compared to individuals over the age of 45 (27). Correspondingly, another study involving 22,085 women in China unveiled that the younger group, specifically those aged 20 to 30, is more susceptible to sensitive or highly SS in comparison to the elder group, aged 30 and above (28). These findings suggest a plausible mechanism wherein the nerve innervation function in the human body, related to touch and pain, diminishes as individual age (29).

### 3.2 Gender

Extensive empirical studies have consistently shown that women are more prone to SS than men. Notably, a study based on 3,012 participants in India found that the proportion of women experiencing “sensitive” or “very sensitive” skin is significantly higher (36.7%) compared to the men (27.9%) (30). These findings are consistent with multiple investigations conducted domestically and internationally, which consistently demonstrated that women are more likely to develop SS than men (23, 31, 32). One plausible explanation for this disparity could be the significant differences in epidermis and dermis thickness between males and females (33), with males exhibiting a greater thickness. Consequently, the skin of males is less permeable than that of females, making it less susceptible to the effects of irritants or allergens. Additionally, the periodic hormonal fluctuations experienced by women contribute to increased SS (34).

### 3.3 Race

While no significant racial differences were observed in the prevalence of SS, variances in sensory perception among different races have been documented. For instance, European Americans tend to exhibit heightened sensitivity to wind, Asians are more sensitive to spicy food, and Hispanics display relatively lower responsiveness to alcohol (35). There are also reports suggesting that African Americans have a higher incidence of SS compared to European Americans (36). As human skin exhibits individual differences, further parallel studies are required to ascertain the true racial disparities in SS (37).

### 3.4 Occupation

Medical personnel wear masks for extended durations while on duty, and studies have documented skin-related symptoms, such as redness and itching, associated with mask-wearing among medical staff (38). However, a survey revealed a prevalence of SS found that there was no significant difference between the socio-professional categories in SS (39). Hence, further research is required to shed light on the influence of occupation on SS.

## 4 Physiological and biological characteristics

### 4.1 Emotional change

Emotional change has been identified as a contributing factor to the development of SS (32, 34). Individuals with SS are more likely to experience discomfort, such as tingling and itching, during the periods of emotional fluctuation compared to individuals without SS (27). Moreover, there is a significant correlation between the occurrence of SS and anxiety, suggesting that anxiety may play a role in the manifestation of SS symptoms (13). Additionally, depression has been associated with SS, where facial flushing is recognized as the primary clinical manifestation (31).

### 4.2 Skin type

Compared to neutral skin, dry, oily, and mixed skin exhibit varying degrees of damage to the skin barrier function, rendering them more susceptible to SS and inflammatory skin diseases (40, 41). Epidemiological studies have further confirmed that these skin types are risk factors for SS diseases (28, 42).

### 4.3 Sleep disorder

Individuals with poor sleep quality exhibit an increase in transepidermal water loss (TEWL) compared to those with good sleep quality, consequently leading to the damage of skin barrier (43). Sleep disorders contribute to an elevation in oxidative stress, resulting in alterations in skin homeostasis and the disruption of inflammatory pathways, ultimately leading to the dysfunction of the skin barrier (44, 45). Impaired skin barrier function and reduced water content in the stratum corneum are significant pathophysiological characteristics observed in individuals with SS (46). An epidemiological survey conducted across multiple countries, including Brazil, China, France, Russia and the United States, found a higher prevalence of SS among subjects with sleep disorders (47).

### 4.4 Menstrual cycle for female

The presence of estrogen receptors in the epidermis impacts skin hydration and the functionality of skin barrier, as estrogen levels directly influence these factors (48). Muizzuddin’s research demonstrated that the strength and dryness of skin barrier, as well as the sensitivity to lactic acid stinging, vary in response to fluctuations in estrogen levels during the menstrual cycle (49). A questionnaire survey involving Dutch women revealed that approximately 42% of premenopausal women experienced an increased sense of SS prior to and during the menstrual cycle (34).

## 5 Lifestyles

### 5.1 Spicy food

Capsaicin, a biologically active component found abundantly in peppers, interacts with the Capsaicin receptor, a transmembrane channel protein widely expressed in skin tissue (50). The activation of the Capsaicin receptor, known as TRPV1, stimulates the release of neuropeptides and excitatory amino acids from free nerve endings, ultimately leading to the sensation of pain in the cerebral cortex (51). Studies have revealed that individuals of Asian descent exhibit increased susceptibility to SS when exposed to the stimulation of a spicy diet (35). A survey conducted among college students in China further confirmed that a spicy diet serves as a risk factor for SS (32). Moreover, experimental evidence has demonstrated that inhibiting the expression of TRPV1 can be effective in treating SS (52).

### 5.2 Cosmetic usage

Research indicates a positive correlation between the frequency of cosmetic usage and the prevalence of SS (53). Specifically, when women utilize multiple types of cosmetics and makeup removers on a daily basis, it can easily trigger SS (28). Brenaut carried out a meta-analysis of 13 surveys, which collectively identified cosmetics as a common trigger for SS, and excessive or improper usage exacerbating this reaction (54). One potential explanation for this phenomenon is that frequent and simultaneous use of cosmetics may thin the stratum corneum on free ending is compromised, leading to a significant increase in nerve sensation input and signal release, resulting in uncomfortable sensations (55). Additionally, a thinner stratum corneum can enhance the penetration of capsaicin, thereby increasing SS (56).

### 5.3 Smoking

Pavithra carried out a proteomic analysis on primary human keratinocytes exposed to long-term cigarette smoke condensate, revealing that the differentially expressed proteins were predominantly associated with the integrity of the epithelial barrier and the anti-inflammatory response (57). Moreover, excessive smoking over 20 cigarettes per day for at least 2 years can disrupt the homeostasis of the epidermal permeability barrier (58). Studies simulating cigarette smoke exposure have further shown that prolonged and/or repeated exposure to high levels of toxic smoke pollutants can impair the function of the epidermal barrier (59). Multiple epidemiological investigations have also confirmed a positive correlation between smoking and the prevalence of SS (60, 61).

### 5.4 Alcohol consumption

Epidemiological studies have established a link between alcohol consumption and an increase in the prevalence of SS (35). It is believed that the decomposition of ethanol into acetaldehyde induces vasodilation, leading to facial burning and flushing (62, 63).

### 5.5 Physical exercise

Prior research has demonstrated that various stimuli, including pain sensation, may undergo changes during and after exercise, potentially attributed to the activation of exercise-induced opioid substances (64). Limbs exhibit a decrease in skin thermal sensitivity only after high-intensity exercise (65). The tingling, burning and itching symptoms associated with SS are also connected to sensory nerve dysfunction in the skin (66). In addition, research has found that spending more time exercising is associated with a lower risk of developing depression (67), which potentially reducing susceptibility to SS (31).

## 6 Environment features

Environmental features encompass natural elements, which are those aspects of the environment existing without significant human modification or intervention. This category comprises variables such as sunlight exposure, temperature and humidity, air pollutions (68).

### 6.1 Sunshine

A large body of literature has examined the effects of ultraviolet (UV) rays from sunlight on the skin. UV rays are categorized into UVA, UVB, and UVC based on their wavelengths, with UVC being absorbed by the ozone layer (69). UVA primarily impacts the dermis and promotes oxidative damage to DNA, while UVB mainly affects the epidermis (70). Studies have indicated that exposure of human keratinocyte to UVB radiation can disrupt the hydration of stratum corneum (71). Numerous experiments utilizing UVB irradiation on epidermal mice have revealed epidermal barrier dysfunction, including increased epidermal thickness, elevated TEWL, and reduced water content in the stratum corneum (72, 73). Furthermore, UVB radiation induces oxidative damage and triggers inflammatory reactions to keratinocyte on the skin surface (74–76), herein contributing to the development of SS. A survey performed on the Chinese population highlighted the gender and radiation dose-related changes in stratum corneum function caused by ultraviolet radiation (77). Building upon this, Francesca and his collaborators discovered a significant cumulative effect of UV and O_3_ in reducing skin barrier-related proteins, such as silk fibroin and skin protein (78). Epidemiological investigations have confirmed that sunlight represents a significant factor influencing the prevalence of SS (32, 79, 80).

### 6.2 Temperature and humidity

Temperatures exceeding 43°C have been shown to directly activate TRPV1 (81). TRPV1 is a specific member of the transient receptor potential (TRP) superfamily of ion channels. Which are found in various tissues throughout the body. TRPV1 leading to the sensation of heat and pain (50). Research has found that the expression of TRPV1 in SS is enhanced, leading to overload of harmful stimuli (82). Therefore, patients are prone to experiencing abnormal heat and pain sensations. In the case of SS, inflammatory mediators can significantly lower the activation threshold of TRPV1 (83). Epidemiological studies have identified changes in temperature and humidity as triggering factors for SS (54, 80). Individuals with SS are particularly sensitive to TEWL and ambient temperature (84). Research has demonstrated that low humidity is more likely to induce SS (23, 54).

### 6.3 Air pollution

The prevalence of SS has been associated with air pollution (22, 85). Among air pollutants, fine particulate matter (PM_2.5_) has been extensively studied. PM_2.5_ exposure in human keratinocytes has been shown to increase Cyclooxygenase2 (COX-2)/Prostaglandin E2 (PGE2) levels, leading to the downregulation of expression of silk fibroin (86, 87). Treatment of human and mouse skin equivalents with PM_2.5_ has also been found to inhibit the expression of fibronectin and increase TEWL, thus compromising the integrity of the skin barrier (88). Additionally, PM_2.5_ can disrupt the morphology and structure of keratinocyte, further compromising the skin barrier (89). Previously, it has been found that exposure to nitrogen dioxide (NO_2_) increases the TEWL value of skin, leading to damage to the skin barrier (90). Low concentration of carbon monoxide (CO) can inhibit the release of tumor necrosis factor-α，interleukin-1β and interleukin-10 reducing inflammatory response, therefore CO may reduce damage to the skin barrier (91), It has been shown that ozone (O_3_) exposure will lead to various skin inflammation through redox pathway (92), At the same time, O_3_ therapy can be applied to the clinical treatment of various inflammatory skin (93, 94), but its role in SS needs further research. Air pollution can cause anxiety in people (17), and the prevalence of SS is significantly correlated with anxiety (13).

## 7 Discussion

Environment encompasses natural environment and built environment, the built environment is defined as encompassing all buildings, spaces, and products that are created or modified by people (14). Factors within this domain include population density, the availability of green spaces, access to food stores, and the connectivity of streets (95).

### 7.1 Empirical associations between BEs and NCDs

The growing body of evidence supports the notion that the BEs plays a significant role in development of NCDs through changes in environmental exposure and health-related lifestyles. For instance, individuals residing in densely populated communities often face a higher risk of obesity (96). As reported, a densely populated community are likely to emit more air pollutants (97) that contribute to an increase in risk of obesity (98, 99). Moreover, population density has a positive correlation with PM_10_ concentration (100), and elevated PM_10_ levels have been shown to compromise lung function (101, 102), consequently impacting cardiovascular health (103). Interestingly, research have indicated that an increase in coverage ratio of greenspaces benefits to lower the prevalence of obesity (104), hypertension and cardiovascular disease (105, 106), as well as reduce the mortality of cardiovascular disease (107, 108). This relationship can be attributable for that urban greenness promotes physical activity (109) and mitigates exposure to PM_2.5_ (110). A survey conducted in Apulia found the impact of green space use on depressive symptoms, and the mediating role of perceived social support in the association (111). Nighttime artificial light (ALAN) has emerged as a risk factor for physiological functions, with a significant correlation observed between higher levels of nighttime outdoor light and poor sleep patterns (112). Notably, ALAN is likely to reduce sleep duration (113), which is more prevalent in communities with higher poverty levels (114). Moreover, sleep disturbance may play a role in anxiety and depressive disorders (111). Besides, research studies have demonstrated that the exposure to Artificial Light at Night (ALAN) during adolescence could potentially elevate the risk of developing atopic diseases during youth (115). Further research is warranted to substantiate causal relationships, particularly those related to circadian rhythm disorders and endocrine pathways. Additionally, a high density of road intersections increases the likelihood of walking and cycling (116). Utilizing public transportation necessitates walking or cycling to bus transits, resulting in elevated levels of moderate physical activity (117, 118). As observed, physical inactivity in both adults and children elevates the risk of overweight/obesity (119–121). Conversely, engaging in physical activity helps to decrease the prevalence and mortality of cardiovascular disease (122, 123). Furthermore, a higher density of fast food restaurants has been associated with a greater prevalence of obesity (124) due to the residential surrounding food BEs have potentials to alter residents’ dietary behavior and quality (125, 126), thereby affecting their health.

Overall, the BEs exerts an influence on individual health through changes in exposure to environmental hazardous stressors and health-related lifestyles (25). SS, identified as a common clinical symptom, results from various internal and external factors. Given the close relationships between BEs and individuals’ daily routines, it is plausible that BEs may also have potential impacts on SS.

### 7.2 Theoretical pathways from BEs to SS

**Figure 2:**
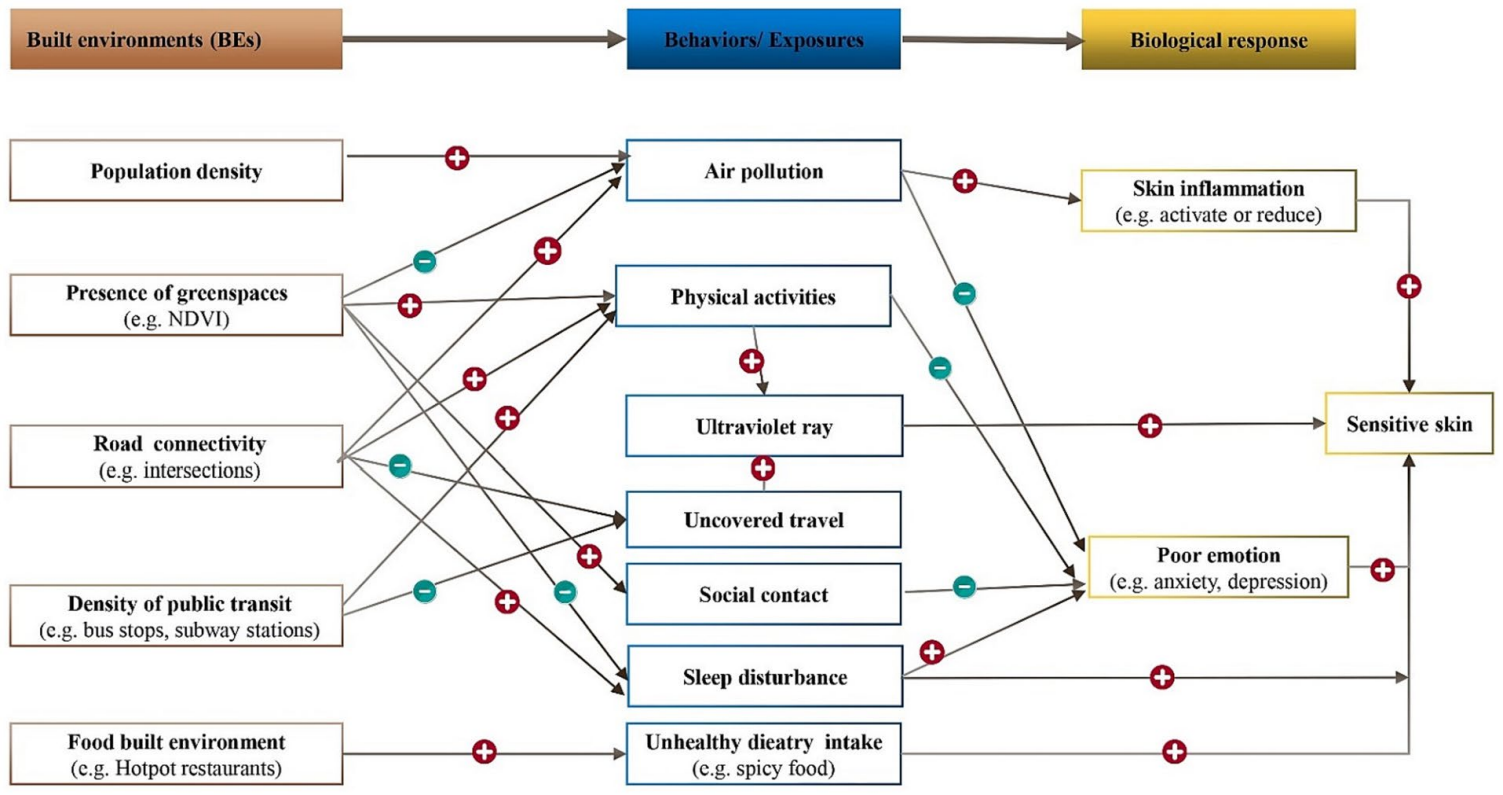
Potential pathways linking BEs to SS. The plus and minus signs in the chart represents positive and negative associations between the two linked variables. NDVI corresponds to the normalized difference vegetation index.

The relationship between the BEs and the development of SS has not yet been fully explored. Based on identified pathways from BEs to other NCDs, our study aims to establish a theoretical framework connecting BEs and SS, providing a scientific foundation for future epidemiological investigations, basic research, and clinical studies. As previously mentioned, SS development may be influenced by factors such as population density, the availability of green space, street design, and the food environment, Additionally, natural environmental exposure and individual behaviors serve as mediating pathways that can contribute to human skin inflammation and the emergence of adverse emotions, ultimately culminating in the manifestation of SS. When skin inflammation occurs, a substantial quantity of inflammatory cytokines and chemokines is released, leading to heightened sensitivity of nerve fibers within the epidermis and an increased responsiveness to external stimuli (127, 128). The association between negative emotions, such as anxiety and depression, and SS has primarily been explored through epidemiological surveys, which have demonstrated a positive correlation. However, there remains a dearth of experimental research evidence on this subject (Figure 2).

#### 7.2.1 Population density

Exposure to PM_2.5_ and emotional change are likely to exhibit mediating role in the association between Population density and SS. High population density has been found to be correlated with an elevated risk of anxiety (129, 130). Notably, anxiety itself has been linked to a higher likelihood of developing SS (13). Furthermore, studies have indicated that as the population density of cities increases, so does the concentration of PM_2.5_ (131). Through experimental verification, researchers have demonstrated that PM_2.5_ can activate the skin’s inflammatory process, resulting in the release of inflammatory factors such as TNF-a and IL-1. This activation can trigger skin nerve fiber sensitivity and impede the repair of the damaged skin barrier (132, 133). Hence, it can be inferred that high population density may contribute to an increased prevalence of SS by elevating anxiety levels and raising PM_2.5_ concentrations.

#### 7.2.2 Green spaces

Urban intervention of greenspace was anticipated to promoting general, mental and physical health. The influence of green spaces on mitigation of the particulate matter concentrations has been extensively researched, revealing various mechanism that are contingent upon environmental and vegetation characteristics (134). A study conducted in Spain involving 39 schools found that higher levels of greenery within and surrounding schools are correlated with reduced levels of traffic-related pollutants (135). Increased levels of ambient particulate matter have been linked to a higher prevalence of SS (136). Moreover, poor emotions such as depression, anxiety, and stress (137) have been recognized as risk factors influencing SS (138). Empirically, urban green spaces have been reported to encourage residents to engage in physical activities (139, 140), which helps to alleviate anxiety (141), thereby protecting against the occurrence of SS. Given the aforementioned association, it is speculated that green spaces could reduce ambient air pollution and increase outdoor activity time, thereby lowering the prevalence of SS through the reduction of exposure to air pollution and alleviation of anxiety levels. In addition, higher tree canopy cover was associated with more favorable sleep (16), so it is speculated that green spaces can reduce SS by improving sleep.

Additionally, a significant statistical correlation has been observed between the proximity of green spaces to residences and the indoor microbial diversity index (142). Exposure to urban green spaces has the potential to increase the diversity of skin microorganisms and alter the composition of human microbiota (143). However, the results from different studies examining the differences in microorganisms between SS patients and normal individuals have been inconsistent (144, 145). Consequently, further research is warranted to ascertain the alterations in microorganisms associated with SS.

#### 7.2.3 Street designs

The density and connectivity of the road network and the availability of public transits play a significant role in influencing residents’ travel mode (146, 147). Although encouraging physical activities, active modes of travel, such as walking and cycling, can increase exposure to ultraviolet radiation, which has been shown to potentially trigger SS conditions. Conversely, motorized modes of travel like busses, self-driving, and subways reduce physical activities but can minimize exposure to ultraviolet radiation. People have experienced higher exposure to air pollutants at intersections (148), and dense intersections have adverse impacts on sleep (149). Therefore, it is speculated that intersections could increase ambient air pollution and sleep disturbance, thereby potentially increasing the prevalence of SS.

#### 7.2.4 Food environment

A spicy diet is a potential risk factor for SS. Health status is associated with the local food environment (150, 151). The proliferation of hotpot and barbecue establishments in residential and workplace areas may contribute to increased consumption of spicy foods, thereby potentially influencing the occurrence of SS. The number of hotpot and barbecue shops serves as an indicator of the built environment, but empirical research is necessary to determine their impact on SS.

## 8 Conclusion

SS, characterized by heightened skin reactivity, is influenced by various internal and external factors. Our study indicates a higher prevalence of SS among women compared to men, with its occurrence diminishing with age. Emotional fluctuations, skin type (dry, oily, or mixed), sleep disturbances, menstrual cycles, consumption of spicy foods, improper cosmetic use, smoking, alcohol consumption, and physical activity also impact SS. Furthermore, environmental factors such as sun exposure, airborne particulate matter, temperature, and humidity contribute significantly. Notably, the connection between the built environment and SS has not been previously explored. Hence, an analysis has been undertaken to summarize the influence of the built environment on individual behavior and the natural environment, with the objective of uncovering a potential indirect connection between the built environment and SS (refer to Figure 2 for comprehensive details).

Health outcomes result from intricate interactions between human biology and the environment. Existing research demonstrates that gene–environment interactions can influence various chronic conditions like obesity (152), cardiovascular diseases (153), and respiratory disorders (154). Recent genome-wide association studies have identified genetic variations in the 2p21 region associated with self-reported SS in the Han population (155). Moreover, Yang et al. reported reduced expression of CLDN5 in the facial skin lesions of SS patients from the Han population compared to unaffected individuals (156). Nonetheless, further scientific validation is required to ascertain the extent to which gene–environment interactions contribute to the development of SS.

The comprehension of how the built environment influences health constitutes an essential component in the development of effective strategies geared toward the promotion of physical and mental well-being. Lifestyle factors, encompassing engagement in sports activities, dietary choices, and stress levels, are all subject to influence by the building environment, and they hold a pivotal role in the management of chronic diseases. The creation of an environment that fosters healthy behavior has the potential to contribute to the management of diseases such as obesity, cardiovascular disease, and skin diseases. The skin, functioning as the interface between the human body and the environment, is notably susceptible to the influences of the built environment. Understanding how building environments impact skin diseases is crucial for comprehending the role of environmental factors in skin health. This knowledge, once acquired, can provide valuable insights for urban planning decisions, inform public health interventions, and guide individual behaviors to cultivate a skin-friendly environment, thereby contributing to the reduction of the prevalence and severity of skin diseases.

This article explores the potential indirect relationship between the built environment and SS by investigating traditional factors influencing SS and the impact of the built environment on individual behavior and the natural surroundings. To establish such associations, a comprehensive approach encompassing geography, public health, and clinical medicine is necessary. We recommend conducting a large-scale epidemiological survey on SS, analyzing epidemiological data, and delving into the pathways and mechanisms through which the built environment influences SS. Ultimately, enhancing the built environment can serve as an effective strategy for the prevention and management of SS.

## Author contributions

XCh: Writing – original draft, Data curation, Formal analysis, Methodology, Supervision, Writing – review & editing. JW: Data curation, Methodology, Writing – review & editing, Formal analysis. WW: Data curation, Formal analysis, Methodology, Writing – review & editing. QP: Methodology, Writing – review & editing. XCu: Methodology, Resources, Supervision, Writing – review & editing. LH: Funding acquisition, Methodology, Resources, Supervision, Writing – review & editing.
