# Supplementary material for: A review of factors influencing sensitive skin: an emphasis on built environment characteristics
Source: Front Public Health. 2023 Dec 4;11:1269314. doi: 10.3389/fpubh.2023.1269314 (PMC10726041; doi:10.3389/fpubh.2023.1269314)
Supplement: Supplementary file 1 [file Table_1.DOCX]

| **Table S1** | **Summary of selected studies between sensitive skin and Socioeconomics Attributes of Population** | | | | | |
| --- | --- | --- | --- | --- | --- | --- |
| **Author and year** | **Location** | **Sample** | **Aim of the study** | **Type of study** | **Statistical method** | **Main results** |
| Misery, L(2018) | France | N=5000 | To investigate the relationship between the age, skin type, quality of life and sensitive skin. | Cross-sectional study | t-test, Chi-square test,wilcoxon test. | Sensitive skin is more common in young people and in women as well as patients with dry skin or fair skin or an atopic predisposition |
| Brenaut, E(2019) | Indian | N=3012 | To evaluate the epidemiology of sensitive skin in the Indian population | Cross-sectional study | t-test, Chi-square test,wilcoxon test. | Women are more susceptible to sensitive skin,climatic factors, environmental factors, cosmetics and food intake were related to sensitive skin |
| Misery, L(2007) | France | N=2007 | To investigate the effects of sensitive skin on quality of life and the psyche, and on seasonal changes. | Cross-sectional study | Analysis of variance, Mann–Whitney U and Kruskal–Wallis H tests,Chi-squared test， | The degree of sensitivity was significantly higher in the female population,Persons with sensitive skin were more numerous in summer than in winter. |
| Jourdain, R(2002) | San Francisco,United States | N=811 | To examine possible ethnic variations in perception of sensitive skin | Cross-sectional study | t-test | The differences in skin sensitivity between ethnic groups concerned mostly factors of skin reactivity |
| Farage, MA(2010) | United States | N=1032 | To compare gender difference with regard to perceptions about sensitive skin. | Cross-sectional study | Chi-square or Mantel-Haenszel (MH) | For sensitive skin of the face and genital area, the perception of skin sensitivity appeared to less severe reactions for the men. |
