# Supplementary material for: A review of factors influencing sensitive skin: an emphasis on built environment characteristics
Source: Front Public Health. 2023 Dec 4;11:1269314. doi: 10.3389/fpubh.2023.1269314 (PMC10726041; doi:10.3389/fpubh.2023.1269314)
Supplement: Supplementary file 2 [file Table_2.DOCX]

|  | **TableS2** | **Summary of selected studies between sensitive skin and Physiological Biology** | | | | |
| --- | --- | --- | --- | --- | --- | --- |
| **Author and year** | **Location** | **Sample** | **Aim of the study** | **Type of study** | **Statistical method** | **Main results** |
| Misery, L | Brazil, China, France Russia, and the United States | N=10743 | To investigate the roles of poorly known associated and triggering factors on sensitive skin in a large global population. | cohort study | t-tests, Mann– Whitney, logistic regres- sion analyses | Female hormonal status, fatigue, sleep disorders and food were associated with sensitive skin |
| Falcone, D(2017) | Netherlands | N=278 | To investigate the perceived influence of fluctuating hormone levels on self-assessed sensitive skin | Cross-sectional study | Fisher’s exact test,Kruskal-Wallis and Mann-Whitney tests | The prevalence of the perceived effects of fluctuating hormone levels on self-assessed sensitive skin in women is high. |
| Kim, YR(2018) | Korea | N= 1000 | To evaluate the prevalence and characteristics of sensitive skin in a Korean population, and compared the results with those of populations from other countries. | Cross-sectional study | t-tests,Wilcoxon and Kruskal- Wallis tests | Sensitive skin group were 2-3 times more reactive to climatic and environmental factors, cosmetics, food items,and emotional status than non-sensitive skin group. |
| Manav, V(2022) | Turkey | N=75 | To investigate the relationship between anxiety levels and facial neurosensitivity, the erythema index, sebum content, and sensitive skin scale scores | Cross-sectional study | Spear- man correlation analysis, Mann- Whitney U test | A strong positive correlation was found between the HADS-Anxiety scores and the erythema index in patients with sensitive skin. |
| Legeas, C(2021) | France | N=160 | To compare subjects with and without symptomatic sensitive skin and to propose diagnostic criteria for sensitive skin | Cross-sectional study | Mann-Whitney U,Pearson’s correlation coefficient | In the sensitive skin group, the subjects more often presented with mixed or dry skin . |
