# Supplementary material for: A review of factors influencing sensitive skin: an emphasis on built environment characteristics
Source: Front Public Health. 2023 Dec 4;11:1269314. doi: 10.3389/fpubh.2023.1269314 (PMC10726041; doi:10.3389/fpubh.2023.1269314)
Supplement: Supplementary file 3 [file Table_3.DOCX]

|  | **Table S3** | **Summary of selected studies between sensitive skin and lifestyles** | | | | |
| --- | --- | --- | --- | --- | --- | --- |
| **Author and year** | **Location** | **Sample** | **Aim of the study** | **Type of study** | **Statistical method** | **Main results** |
| Yasak Guner, R(2022) | Turkey | N=349 | To examine facial Demodex mites density and other factors as- sociated with sensitive skin in patients. | Cross-sectional study | Linear regression analysis and cor- relation analysis | Skin sensitivity increased with more frequent cosmetic use. |
| Xiao, X(2020) | China | N= 22,085 | To investigate the prevalence of sensitive skin at all ages and the impacts of related factors across China. | Cross-sectional study | Analysis of variance,Wilcoxon and Kruskal–Wallis tests | Skin sensitivity is associated with age, skin type, geographic area of subjects, Heavy life stress and the application of several cosmetic products. |
| Brenaut, E(2020) | France |  | To perform a systematic literature review to collect data on the triggering factors involved in SS and to then perform a meta-analysis | Meta-analysis |  | The most important triggering factor of sensitive skin was cosmetics, Other triggering factors were physical, chemical, or psychological factors. |
| Falcone, D(2017) | Netherlands | N=258 | To identify risk factors that increase the likelihood of sensitive skin | Cross-sectional study | Univariate logistic regression,multivariate logistic regression | Atopic predisposition, presence of skin diseases and Fitzpatrick skin types I and II , smoking and a history of low sun exposure are risk factors associated with Sensitive skin. |
| Fawkes, N(2021) | Britain | N=3050 | To identify common inherent and external triggers of sensitive skin. | Cross-sectional study | t-test,Chi-square test,Spear- man correlation analysis | Sensitive skin is related to age, gender, and smoking status |
