# Supplementary material for: A review of factors influencing sensitive skin: an emphasis on built environment characteristics
Source: Front Public Health. 2023 Dec 4;11:1269314. doi: 10.3389/fpubh.2023.1269314 (PMC10726041; doi:10.3389/fpubh.2023.1269314)
Supplement: Supplementary file 4 [file Table_4.DOCX]

|  | **Table S4** | **Summary of selected studies between sensitive skin and natural environmental factors** | | | | |
| --- | --- | --- | --- | --- | --- | --- |
| **Author and year** | **Location** | **Sample** | **Aim of the study** | **Type of study** | **Statistical method** | **Main results** |
| Kamide, R（2013） | Japan | N=1500 | To assess the prevalence and the characteris- tics of sensitive skin in 2011 in Japan | Cross-sectional survey | Analysis of variance,wilcoxon test. | Water, air pollution, and warm climatic conditions were considered the factors related to sensitive skin |
| Ma, L（2016） | ShangHai，China | N=369 | To investigate the prevalence and factors related to the scalp sensitivity in China. | Cross-sectional survey | Analysis of variance ，Chi-squared test，logistic regression analysis | Air dryness, exercises, humidity, heat, and sun were significantly associated with sensitive skin |
| Lan, L（2008） | America | N=994 | To assess the prevalence of sensitive skin and collect data on sensitive skin in the US population. | Cross-sectional survey | t-test, Chi-square test,wilcoxon test. | Sensitive skin is mainly associated with dry skin, fair phototype, reactivity to climatic and environmental factors, and cosmetics |
| Vanoosthuyze, K（2013） | European and America | N=1831 | To evaluate dermatologists’ perceptions with regard to the prevalence of sensitive skin among men, and the potential impact of influenc- ing factors | Cross-sectional survey | t-test | Environmental factors（exposure to UV light）, stress, increased acceptance in society and increased use of products are important for men with sensitive skin. |
| Brenaut, E(2020) | France |  | To perform a systematic literature review to collect data on the triggering factors involved in SS and to then perform a meta-analysis | Meta-analysis |  | The factors were cosmetics, wet air, air conditioning, heat, and water. |
| Feng, Y(2021) | Xinjiang,  China | N= 3584 | To characterize the SS facial skin in normal adolescents of Xinjiang. | Cross-sectional survey | t-test, Chi-square,Multiple linear regression. | Sun exposure，spicy food, mood change, and skin care products ， family history are contributed to facial sensitive skin. |
| Wang, X(2020) | Guangzhou,  China | N=956 | To characterize facial sensitive skin in normal young Chinese | Cross-sectional survey | t-test,Chi-square test,Fisher’s exact test | Low humidity and sun exposure, emotional factors were main factors affecting sensitive skin. |
