# Supplementary material for: A review of factors influencing sensitive skin: an emphasis on built environment characteristics
Source: Front Public Health. 2023 Dec 4;11:1269314. doi: 10.3389/fpubh.2023.1269314 (PMC10726041; doi:10.3389/fpubh.2023.1269314)
Supplement: Supplementary file 5 [file Table_5.DOCX]

| **Table S5** | **Summary of selected studies between natural environment, Personal behavior and built environment** | | | | | |
| --- | --- | --- | --- | --- | --- | --- |
| **Author and year** | **Location** | **Sample** | **Aim of the study** | **Type of study** | **statistical method** | **Main results** |
| Tao, Z(2016) | China | N=1048 | To predictive nature of crowded living conditions on binge eating and the use of the Internet as coping strategies. | Cross-sectional study | Multivariable regression | Among female participants, binge eating scores were significantly predicted by anxiety caused by high-density living conditions. Among male participants, the binge eating scores significantly predicted by the anxiety caused by high-density living conditions and self-control. |
| Chan, SM(2021) | HongKong,  China | N=1978 | To enrich the comprehension of the effect of living density on anxiety and stress among adults in a global city | Cross-sectional study | Logistic regressions | The living density was significantly associated with anxiety and stress of residents. female, younger adults or those living in income poverty were also at risk of anxiety and stress. |
| Kim, MJ(2021) | Korea | N=254 cities | To analyze the impact of three major socio-economic factors on PM 2.5 concentrations for 254 cities. | Panel study | The multivariate panel regression | Income,density, and population are associated to PM 2.5 pollutions. |
| Dadvand, P（2015） | Barcelona, Spain | N=37 schools | Investigated the association between greenness within and surrounding school boundaries and monitored indoor and outdoor levels of traffic-related air pollutants. | Cross-sectional study | Mixed effects models | Higher greenness within and surrounding school boundaries was consistently associated with lower indoor and outdoor traffic-related air pollutants levels. |
| Yuen, JWM（2019） | Hong Kong, China | N=554 | To understand how green space and accessibility of common public open spaces in compact urban areas affect physical activity and healthy diets of residents. | Cross-sectional study | Analysis of variance，Chi-square test,Spear- man correlation analysis | With the increase of green spaces, residents will engage in more moderate to high intensity physical activities |
| Beyer, KM（2014） | America | N= 2,479 | To examine the relationships between green space and mental health | Cross-sectional study | Multivariate linear regression | Higher levels of neighborhood green space were associated with significantly lower levels of symptomology for depression, anxiety and stress. |
| Li, H(2022) | Chengdu; China | N= 638 | To understand how the rural built environmen have impacted rural children’s school travel mode choice | Cross-sectional study | The multinomial logit model | The improvements in road layout and facility conditions are significantly and positively associated with children’s choice of electric bicycles for school. |
| Wang, W(2021) | Zhongshan;China | N=4784 | To describes the nonlinear associations of the built environment with cycling frequency among older adults. | Cross-sectional study | XGBoost Model | The intersection density is inversely related to the cycling frequency among older adults. |
| Wang, Y(2019) | China | N=338 cites | To quantify the socio-economic driving factors of PM2.5 concentration changes. | Panel study | Spatial Regression Model | Population agglomeration, industrial development, foreign investment, transportation, and pollution emissions contributed to the increase of PM2.5 concentration. |
| Huang, YJ(2022) | Taiwan; China | N=407,415 | To explore the contributions of green space and ambient PM2.5 to the risk of specific cancers | Retrospective longitudinal cohort study | Cox proportional hazards models | Long-term PM 2.5 exposure is associated with an increased risk of some types of cancers. |
| Paksarian, D(2020) | America | N= 10 123 | To estimate associations of outdoor artificial light at night (ALAN) with sleep patterns and past-year mental disorder among US adolescents. | Cross-sectional study | Binary and multinomial logistic regression | ALAN was associated with less favorable sleep pattern sand mood and anxiety disorder in adolescents. |
| Ohayon, MM(2016) | America | N=19,136 | To explore the associations between outdoor nighttime lights (ONL) and sleep patterns in the human population. | Cross-sectional study | Logistic regression | Living in areas with greater outdoor nighttime lights was associated with delayed bedtime and wake up time ,shorter sleep duration, and increased daytime sleepiness. |
| Xiao, Q(2020) | America | N= 333,365 | To assesse the relationship between outdoor ALAN and self-reported sleep duration | Cross-sectional study | Multinomial logistic regression | The burden of short sleep may be higher among residents in areas with higher levels of outdoor LAN, and this association is likely stronger in poorer neighborhoods. |
| Djurhuus, S(2014) | Denmark | N=28,928 | To examines the associations between objective measures of access to public transportation and self-reported active commuting. | Cross-sectional study | Multilevel logistic regression | Distance to bus stop, density of bus stops, and number of transport modes were all positively associated with being an active commuter and with meeting recommendations of physical activity. |
| Villanueva, K(2008) | Australia | N=103 | To compare pedometer steps of university students who used public transport and private motor vehicles to travel to and or from The University of Western Australia. | Cross-sectional study | t-tests; Chi-square;Multivariate logistic regression | Compared with private motor vehicle users, public transport users performed more daily steps. |
| Choi, H（2018） | South Korean | N=232 administrative | To assess the association between the number of days the accepted PM10 and CO thresholds were exceeded and the concentration of potential emission sources in winter season 2015. | Cross-sectional study | Negative binomial regression | Compared to the districts with the lowest population density, the districts with the third highest population density associated most strongly with air pollution |
